# Paternal Benzo[a]pyrene Exposure Modulates MicroRNA Expression Patterns in the Developing Mouse Embryo

**DOI:** 10.1155/2012/407431

**Published:** 2012-04-04

**Authors:** Asgeir Brevik, Birgitte Lindeman, Gunnar Brunborg, Nur Duale

**Affiliations:** Division of Environmental Medicine, Department of Chemical Toxicology, Norwegian Institute of Public Health, P.O. Box 4404 Nydalen, 0403 OSLO, Norway

## Abstract

Little attention has been given to how microRNA expression is affected by environmental contaminants exposure. We investigate the effects of paternal exposure to benzo[a]pyrene (B[a]P) on miRNA expression in the developing mouse embryo. Male mice were exposed to B[a]P (150 mg/kg i.p.), and their sperm was used four days later in *in-vitro* fertilization experiments. Twenty embryos each from 2-, 8-cell and the blastocyst stage were used for genome-wide miRNA expression profiling. Paternal exposure to B[a]P affected the expression of several miRNAs, and the target genes for some of the dysregulated miRNAs were enriched in many different pathways that are likely to be relevant for the developing mouse embryo. By linking the miRNA target genes to publicly available databases, we identified some miRNA target genes that may serve as global markers of B[a]P-mediated genotoxic stress. The dysregulated miRNAs may provide valuable knowledge about potential transgenerational effects of sublethal exposure to chemicals.

## 1. Introduction

Reduced sperm count and sperm quality are reported from many developed economies [1] and there are also increased rates of testicular cancer manifested in Western and Northern Europe [2, 3], Australia, and Northern America [4]. It has been suggested that this negative development could be caused by increased exposure to environmental contaminants. Physical as well as chemical exposures have been associated with reduced sperm quality in association studies [5–10].

Chemical environmental contaminants have been shown to negatively affect reproduction and embryo development in animals [11–15]. In humans, spermatozoa from infertile men demonstrate higher levels of DNA damage compared to fertile men, and sperm DNA damage is associated with low sperm quality [16–19] and reduced fertility [20]. Concern is being raised over the possibility that paternal germ cell DNA damage in humans, induced by environmental contaminants, could have an impact on the next generation.

Benzo[a]pyrene B[a]P is a carcinogenic contaminant with ubiquitous distribution and potential reprotoxic effects [21–25]. B[a]P is found in coal tar, in automobile exhaust fumes (especially from diesel engines), in all smoke resulting from the combustion of organic material (including cigarette smoke), and in charbroiled food. This compound is the chemical compound whose ability to form DNA adducts has been best characterized. B[a]P undergoes metabolic transformation to a diol-epoxide, BDPE, in the human organism [26, 27]. The global distribution and DNA damage-inducing properties of B[a]P make it a relevant genotoxic model compound for the study of potential transgenerational effects of paternal exposure.

MicroRNAs (miRNAs), discovered in 1993, are short (17–25 nucleotides) noncoding RNAs which negatively regulate specific target genes by mRNA degradation or translational repression [28]. miRNAs have fundamental roles in multiple cellular processes and are also implicated in the development of multiple diseases (for a review see [29]). Their importance is evident from phenotypes of knockout and mutant mice and from studies comparing expression profiles. Representing promising therapeutic targets and candidate biomarkers in pathophysiology, miRNAs are an active area of research. Several studies implicate miRNAs in the control of early embryonic development and maintenance of the pluripotent stem cell state [30], but the impact of environmental contaminants on miRNA expression has been little studied so far. Recently, epigenetic mechanisms through which paternal influence on offspring development have received more attention [31], and miRNAs play a key role in epigenetic regulation.

Following paternal acute exposure to B[a]P four days prior to fertilization, we studied the global miRNA expression profile of the developing mouse embryo. We demonstrate that genome-wide miRNA expression profiling studies can be performed on a very limited number of cells and that early embryonic transcription of multiple miRNAs is affected by B[a]P exposure of the fertilizing sperm. To our knowledge, this is the first report on embryonic miRNA modulation, following paternal exposure to environmental contaminants.

## 2. Methods

### 2.1. The Exposure of Male Mice from which Sperm Was Derived for IVF

Exposed males (strain B6D2F1 from Charles River Laboratories, 8–12 weeks of age) received one i.p. injection of B[a]P (150 mg/kg body weight) dissolved in corn oil four days prior to the IVF experiment. Timing of the exposure to B[a]P was based on pilot studies and knowledge about the most susceptible stage of spermatogenesis with respect to dominant lethal mutations. Similarly, aged control males received an equivalent volume of corn oil. At the day of the IVF experiment, males were killed by cervical dislocation. Cauda was surgically removed and collected in an eppendorf tube containing M2 medium (500 *μ*L, Sigma). Using a pair of microscissors, a few incisions were made in the cauda and the sperm was allowed to disperse for 10 minutes in a small drop (250 *μ*L) of HTF medium (EmbryoMax, Millipore) under liquid paraffin (MediCult) before transfer to the IVF dishes. Experiments are based on oocytes from 36 females and sperm from 6 males (3 exposed and 3 controls) altogether.

### 2.2. Superovulation and *In-Vitro* Fertilization (IVF)

Females (strain B6D2F1 from Charles River Laboratories, 4–6 weeks of age) were injected i.p. with pregnant mare serum hormone gonadotropine (PMSG, Folligon from Intervet) (5 IU) three days prior to the IVF procedure. Two days later (i.e., the day before the IVF) animals received an additional i.p. injection of human chorionic gonadotropine (HCG, Ovitrelle from Serono) (5 IU). Mice were killed by cervical dislocation and oviducts were collected in M2 medium (Sigma). Egg clutches (10–20 oocytes) embedded in cumulus cells were extracted from each oviduct. Oocytes were transferred to IVFdishes and incubated in a droplet of HTF sperm containing medium under liquid paraffin for 4.5 h (37°C). Oocytes from one side of the animal were combined with sperm from B[a]P exposed animals and oocytes from the other side where combined with sperm from control animals. Hence, oocytes from all animals were present in both the control group and the exposed group. After 4.5 h, the fertilized oocytes (zygotes) were washed 5X in KSOM medium (EmbryoMax Millipore) before they were transferred to a drop of KSOM (200 *μ*L) in a petri dish (35 mm) under liquid paraffin (MediCult). The zygotes were allowed to grow for harvesting at the 2-cell, 8-cell and blastocyst stage. At harvest, 20 embryos were collected in microtubes filled with 5 *μ*L lysis medium (CelluLyser, TATAA) and then frozen at −80°C until use. Hence, the 20 embryos contained in one sample represent a random selection of fertilized oocytes from 12 different females, all of which had been fertilized by the same male. There are no technical replicates; values are based on one pooled sample of 20 biological replicates at each stage and treatment. 

### 2.3. Genome-Wide miRNA Expression Profiling

Embryo lysates were subjected to global miRNA preamplification using SBI small RNA amplification kit according to manufacture's protocol (System Biosciences (SBI), Mountain View, CA). The Small RNA amplification system consists of three steps: (1) ligation of an adapter to the 3′ end of the RNA, (2) reverse transcription of the RNA and attachment of a 5′-end adapter in the same reaction by template switching, and (3) PCR amplification of the cDNA with a PCR polymerase mixture that incorporates a proof reading function. Brief description of the protocol: in the adapter ligation step RNase-free water (2 *μ*L), SBI ligase buffer (5 *μ*L), SBI 3′ adaptor (0.5 *μ*L), total RNA from lysed samples (5 *μ*L), and SBI ligase cocktail (0.5 *μ*L) were mixed and incubated (1 hour, 37°C). In the first strand synthesis RNase-free water (4 *μ*L), SBI 3′ adaptor primer (0.5 *μ*L) and ligation product from the adaptor ligation (1 *μ*L) were mixed and incubated in two steps (1 minute at 65°C followed by 5 min at 42°C). In the reverse transcriptase step, a mastermix consisting of SBI 5X reverse transcriptase buffer (2 *μ*L), SBI dNTP mix (1 *μ*L), SBI 5′ adaptor (0.5 *μ*L), SBI dithiothreitol (0.5 *μ*L), and SBI reverse transcriptase (0.5 *μ*L) were mixed with the preceding first-strand synthesis product and allowed to incubate in two steps (5 min at 42°C, and then 10 min at 95°C). Reactions were then kept on ice until second-strand synthesis and amplification. In the second strand synthesis and amplification step, the product from the first strand cDNA synthesis step was mixed with RNase-free water (77 *μ*L), SBI 10X PCR buffer (10 *μ*L), SBI dNTP mix (2 *μ*L), SBI 3′adaptor primer (4 *μ*L), SBI 5′adaptor primer (4 *μ*L), and SBI PCR polymerase (3 *μ*L). The reactions were transferred to a thermal cycler (Eppendorf mastercycler) and underwent the following program: 5 min at 95°C, 42 times (25 sec at 95°C, 20 sec at 55°C, 30 sec at 72°C), 30 sec at 72°C, and held at 15°C.

### 2.4. Real-Time qPCR Reaction

Following miRNA amplification, genome-wide qPCR miRNA expression profiling was performed using mouse miRome microRNA qPCR Profiler from System Biosciences, which simultaneously profiles all known mouse miRNAs, on an Applied Biosystems 7500 Real-time PCR system (Applied Biosystems, Foster City, CA). The miRNA profiling was performed by qPCR using microRNA-specific primers and SYBR Green RT2 ROX mastermix (Qiagen, Germany). In brief, each 20 *μ*L qPCR reaction included SYBR Green RT2 ROX master mix buffer (10 *μ*L), SBI universal reverse primer (0.10 *μ*L), globally amplified miRNA samples (0.13 *μ*L), SBI Quantimir microRNA primer (4 *μ*L), and RNase-free water (5.75 *μ*L). The PCR program was 2 min at 50°C, 10 min at 95°C, 40 times (15 sec at 95°C, 1 min at 60°C, 35 sec at 72°C (data read)), followed by a melting curve analysis step.

### 2.5. Data Analysis

Raw *C*_*T*_-values from 457 miRNAs were preprocessed to remove outliers and miRNAs for which there were inadequate measurements. The *C*_*T*_ values equal to 35 cycles were considered as a limit of detection and all *C*_*T*_ values > 35 were removed from downstream analysis. In addition, filtering criterium for missing values was set to 80%, which is the minimum percentage of existing values, and all the patterns with less than 80% existing values were removed. Only those miRNAs passing quality assurance criteria were included in the downstream analysis. Missing values were imputed by average nonmissing values of that particular sample.

The raw *C*_*T*_-values were normalized using the mean expression value of all expressed miRNAs as previously [32, 33], and the mean expression value for individual sample was calculated from miRNAs with *C*_*T*_-values ≤ 35 cycles. The relative expression levels in samples were analyzed by the comparative *C*_*T*_-method [34, 35]. The fold change indicates the expression level of miRNAs from paternal B[a]P exposed samples relative to that of untreated control samples. The miRNA expression levels are hence always relative to the untreated control sample. The fold change values were log2-transformed in order to make the data symmetrical around zero. Unsupervised hierarchical clustering analysis was performed to cluster variables into groups based on their similarity, and the results were visualized in a dendrogram using MeV v4.7 software [36] or J-Express v2009 (MolMine, Bergen, Norway) [37]. The miRWalk database was used to identify potential miRNA target genes [38]. The miRWalk algorithm is based on a computational approach which identifies the longest consecutive complementary between an miRNA and a gene sequences; miRWalk compares the gene's identified miRNA binding sites with the results of eight established miRNA-target prediction programs. The miRWalk database can provide miRNA targets interaction information produced by eight different established miRNAs prediction programs.

For functional enrichment analysis of the miRWalk predicted target genes, we used the WebGestalt V2 [39]. The WebGestalt V2 is a web-based gene set analysis tool, and it is a suite of tools for functional enrichment analysis in various biological contexts. WebGestalt compares a user-uploaded gene list with genes in predefined functional categories to identify those categories with enriched numbers of user-uploaded genes. The WebGestalt V2 enrichments analysis is based on hypergeometric statistical tests, including Benjamini and Hochberg multiple test adjustment. The minimum number of genes per category was set to 2 (default), and the whole mouse genome was used as reference gene set. The enrichment analysis identified the top 10 pathways with the most significant *P*-values.

## 3. Results

Zygotes and embryos from three preimplantation stages (2- and 8-cell as well as the blastocyst stage), fertilized either with control sperm or with sperm from B[a]P-treated mice, were used to study the effect of paternal exposure to B[a]P on the expression of a panel of 456 mouse miRNAs.

A total of 60 *in*-*vitro* fertilized embryos (30 derived from B[a]P exposed sperm and 30 from control sperm) at three different developmental stages were used for the final analysis. We timed our sample extraction according to 24 h cycles, with the 2-, 8-cell, and blastocyst time points referring to 24 h, 72 h, and 120 h, respectively. The stages can be readily identified up to the 8-cell stage with careful visual inspection serving as additional quality criteria upon sampling. Samples were selected exclusively from the pool of healthy looking embryos representative of that particular stage. It is well known that embryonic cell cycle duration is subject to considerable variation, not only among different embryos, but also among blastomeres within the same embryo [40–42].

### 3.1. miRNA Expression Profiling

**Figure 1:**
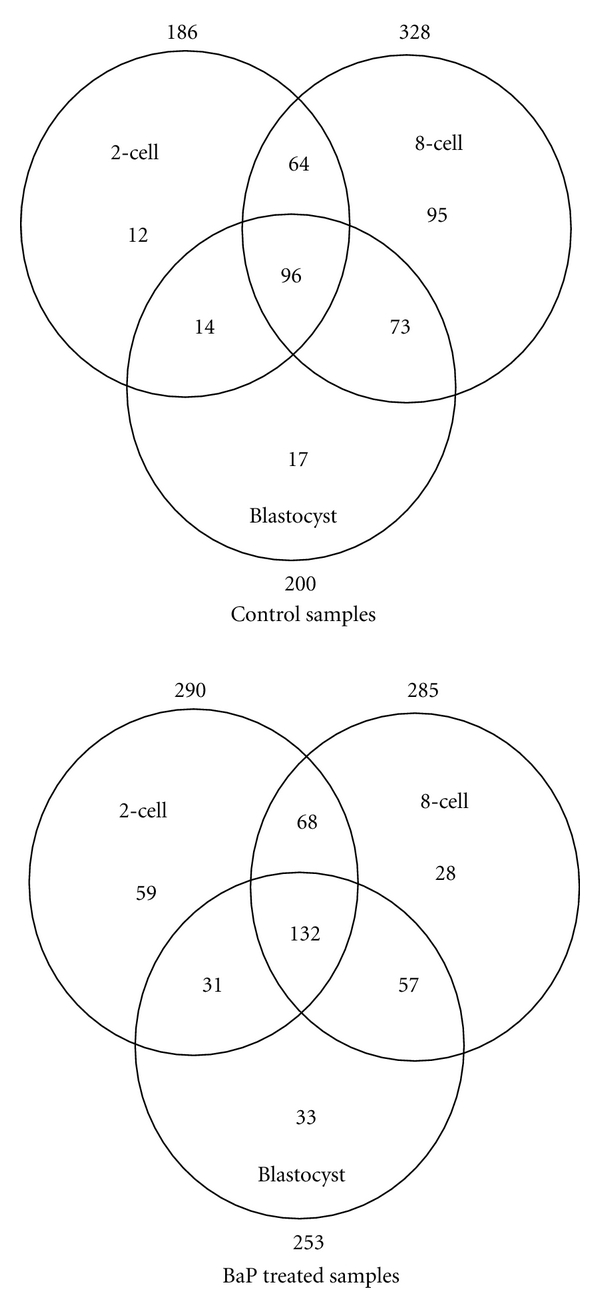
Venn-diagram showing number of samples at the three different developmental stages among control embryos and embryos of B[a]P exposed fathers, respectively.

Figure 1 shows a Venn-diagram of the number of miRNAs expressed at different stages. Some differences were noted in the number of miRNAs expressed at different stages among control embryos and embryos of B[a]P exposed fathers; more miRNAs were expressed in the exposed group at the 2-cell and the blastocyst stage than in controls. At the 8-cell stage, there were more miRNAs expressed in the control group than in the exposed group. The Venn-diagram in Figure 1 also reveals that more miRNAs were expressed consistently across all stages among embryos of B[a]P exposed fathers compared to controls.

**Figure 2:**
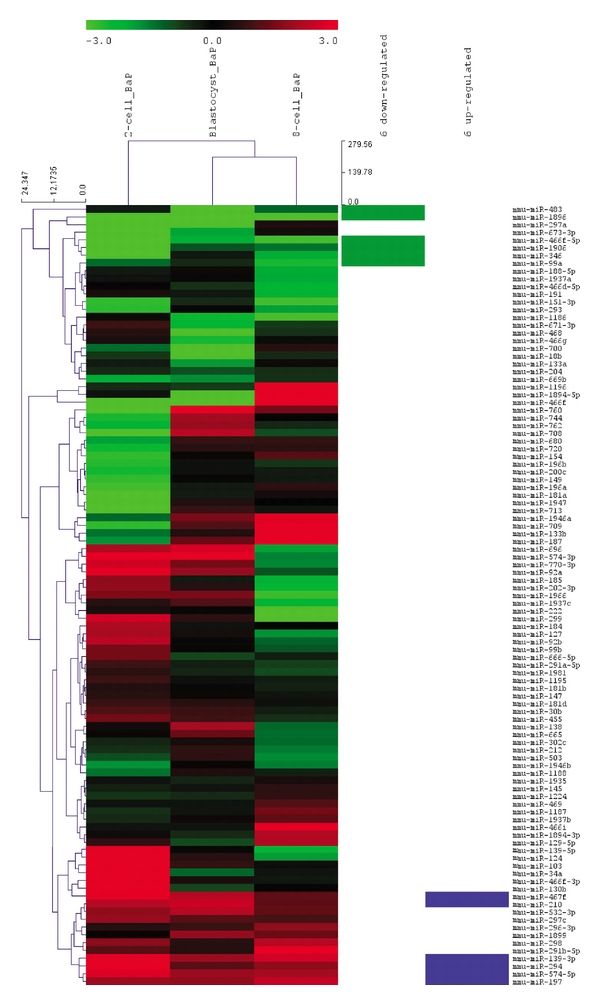
Unsupervised hierarchical clustering analysis of the relative expression of 102 miRNAs after filtering and normalization of the data. The hierarchical clustering analysis is based on similarities in gene expression. Red samples represent upregulated miRNAs whereas green samples represent downregulated miRNAs. The blue color code represents six upregulated miRNAs, and the purple color code represents six downregulated miRNAs, which were selected for further analysis.

The outcome of the quality assurance filtering criteria mentioned above was a set of 102 miRNAs, and these miRNAs were used in the downstream analysis. Unsupervised hierarchical clustering analysis of these 102 miRNAs was performed using MeV v4.7 software [36], and the resulting heatmap is presented in Figure 2. By visual inspection of the heatmap, we observed that the 8-cell and blastocyst embryos were clustered close to each other and shared similar expression pattern compared to the 2-cell embryo (Figure 2). There were also some miRNAs which were predominantly up- or downregulated following paternal B[a]P exposure in all three stages. From these dysregulated miRNAs, we selected six miRNAs from the upregulated part and six from the downregulated part of the spectrum for target-genes analysis (Figure 2). We were primarily interested in B[a]P-induced effects, and therefore we consistently selected the up- or downregulated miRNAs across all stages. The six up- and six downregulated miRNAs selected are shown in Table 1.

We then used the miRWalk database [38] in order to identify potential miRNA targets for the selected B[a]P dysregulated miRNAs. The intersection of identified target genes from at least five prediction programs was chosen, and the numbers of predicted target genes for each selected miRNA are shown in Table 1.

Each miRNA can regulate numerous target genes and therefore has the potential to alter multiple biochemical pathways. To investigate what pathways may be regulated by our twelve selected dysregulated miRNAs, we evaluated the biological functions of their predicted target genes using WebGestalt V2 [39]. We correlated predicted target genes with the KEGG (Kyoto Encyclopedia of Genes and Genomes) biochemical pathways [43] in order to identify enriched pathways. The results from enrichment analysis represent a global picture of pathways that are significantly enriched with target genes for dysregulated miRNAs following paternal B[a]P exposure.

Significantly enriched KEGG pathways annotating the predicted target genes for the twelve dysregulated miRNAs following paternal B[a]P exposure are presented in Table 2. The complete set of significantly enriched pathways annotating target genes are listed in Supplementary Table 1 (see Supplementary Material available at doi:10.1155/2012/407431). Examples of some significantly enriched pathways are cytokine-cytokine receptor interaction, insulin signaling pathway, regulation of actin cytoskeleton, apoptosis, and MAPK signaling pathway; these are shown in Table 2. The identified pathways are enriched mainly with target gene sets for the six upregulated miRNAs following paternal B[a]P exposure. KEGG pathways significantly enriched in target gene sets for the six downregulated miRNAs are involved in cell cycle, neuroactive ligand-receptor interaction, TGF-beta signaling pathway, calcium signaling pathway and chemokine signaling pathway (Table 2). Some pathways were enriched in both the up- and the downregulated target genes.

### 3.2. In-Silico Analysis of the Predicted Target Genes for the Twelve Dysregulated miRNAs

**Figure 3:**
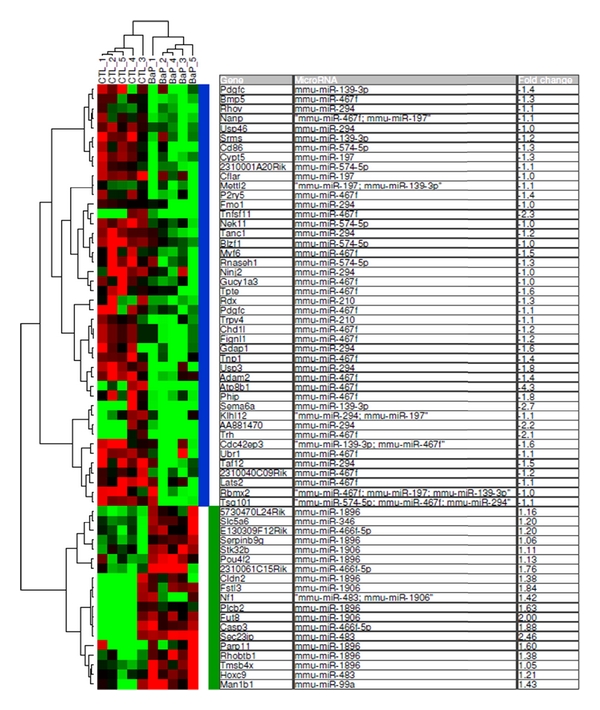
Heatmap showing the result of unsupervised hierarchical clustering of genes from previously published B[a]P sensitive testis dataset (63 genes), targeted by the 12 dysregulated miRNAs identified in the present report. Vertical blue color bar indicates target genes (*n* = 19) for the six upregulated miRNAs, and the green bar indicates target genes (*n* = 44) from downregulated target miRNAs. Average fold change in gene expression is indicated in the far right column. Some of the genes are targeted by more than one miRNA.

We wanted to investigate how the expression pattern of our target genes are modulated in other reported systems in which B[a]P-induced gene expression modulations has been studied. To this end, we performed a search in the publicly available gene expression databases, such as NCBI Gene Expression Omnibus (GEO) [44] and ArrayExpress [45]. Here, we wanted to identify genes from our target gene list that have been modulated by B[a]P exposure to male mice in other systems and at the same time see if the expression pattern of these genes anticorrelate with the expression pattern of the twelve dysregulated miRNAs in our study. Since mRNA expression profiling data from experiments similar to our study are not available, we used the microarray gene expression data from Verhofstad and coworkers [46], represensting a study of gene expression analysis in mice testis following one acute B[a]P exposure (13 mg/kg bw) by oral gavage [46]. Spermatogenesis takes place in testis, and comparing gene expression patterns between the two types of experiments might be useful. Verhofstad and coworkers studied B[a]P exposure-induced changes in gene expression patterns in testis in both wild-type and Xpc-knockout mice. In order to compare our target gene list with the testis gene expression microarray data, we downloaded the raw microarray data (GEO accession number: GSE17979) deposited in the GEO database [44]. These microarray data (the gpr-file: GenePix Results file) were reanalyzed as described previously [47]; all data processing was performed in J-Express v2009 [37]. Only the microarray data from wild-type mice exposed with B[a]P and untreated controls were used for reanalysis. The log2-transformed ratios of the processed intensities were further analyzed by SAM (Significance Analysis of Microarray) [48]. SAM analysis was conducted in order to identify genes whose mean expression level is significantly different between B[a]P exposed and untreated control samples. This resulted in 345 genes identified as significantly differentially expressed (FDR < 10%). We then conducted similarity searches between our target gene list and SAM-identified gene list; this process identified 63 genes which showed anticorrelated expression pattern to our dysregulated twelve miRNAs. Of those genes, 44 were downregulated and 19 were upregulated in mice testis following B[a]P-exposure.  Figure 3 shows hierarchical clustering analysis of these 63 genes. By visual inspection of the heatmap (Figure 3), we observed that B[a]P-exposed samples clustered close to each other in one branch while untreated control samples clustered in the other branch. The gene expression profiles constituted by these altered genes along with their anticorrelated miRNA can be used as a basis for identification of B[a]P-exposure gene expression signatures. Using the results of both miRNA and mRNA expression profiling—in combination with what is known about miRNA functions—we expect that identification of anticorrelated miRNA-mRNA pairs will narrow down the target gene list and refine the predicted miRNA-mRNA interactions.

We also compared our twelve most dysregulated genes with the miRz miRNA expression database [49]. The result of this comparison is expressed in Supplementary Figure 1. It appears, based on the miRz database, that some of the twelve dysregulated miRNAs, most notably miR-210, miR-294, and miRs-466f-5p are highly expressed in several tissues, whereas others (miR-139-3p, miR-197, miR-1896, miR-346, and miR-1906) have not been detected. 

## 4. Discussion

The most important finding from this work is that multiple miRNAs are differently expressed in embryos of B[a]P exposed fathers relative to control embryos. Our results also demonstrate that miRNAs expression can be measured in very few cells. The functional implications of early xenobiotic induced suppressed or enhanced single miRNA expression profiles in developing embryos are not well understood; however, given the broad array of gene transcripts targeted by miRNAs, the perturbations are likely, at some level, to be detrimental to the development of the embryo.

The highly dynamic physiology of the developing embryo is reflected in dynamic miRNA expression profiles. Maternally inherited miRNAs are abundant in the mouse zygote, but many of the maternal gene products are quickly downregulated during the maternal-zygotic transition; some are downregulated by as much as 95% between the 1- and 2-cell stages [50]. A 60% decrease in miRNA expression levels has been reported, indicating that miRNAs may be degraded similar to maternal mRNAs [51]. Transcription of zygotic RNAs begins at the 2-cell stage [52], and ~2.2-fold increases in miRNA expression have been noted from the 2- to 4-cell stage [51]. In the cluster analysis (Figure 2), the 8-cell stage and the blastocyst stage cluster together, indicating that the 2-cell stage exhibited a somewhat different expression profile from the two other stages. The maternal-zygotic transition may represent a particularly sensitive stage of embryonic development.

The predicted target genes for miRNAs that were found to be dysregulated in the present study by paternal B[a]P exposure participate in numerous biochemical pathways. Among the enriched KEGG pathways were those related to metabolism, cancer, cell cycle, apoptosis, MAPK signaling, cytokine-cytokine receptor interaction, and TGF-beta signaling (Table 2 and Supplementary Table 1). The predicted genes affected by the B[a]P-induced miRNA aberrations overlap with previously published B[a]P-sensitive testis gene dataset; the resulting genes are shown in Figure 3. We did not have any information available on embryonic expression of miRNA target genes in our current study. The testis dataset from Verhofstad represents the closest match we could find in the literature, but it is not known to what extent gene expression in the B[a]P exposed developing embryos would match the testis gene expression of B[a]P exposed mice; we are pursuing this question.

Perhaps the most interesting miRNA for embryonic development is mmu-miR-294, a member of the miR-290 cluster. In mouse embryonic stem cells (ES) miR-294 has been shown to promote pluripotency by regulating a subset of c-Myc target genes and upregulating pluripotency-associated genes such as Lin28 [30]. It has been estimated that the miR-290 cluster alone makes up greater than 70% of the total quantity of miRNAs in ES [53]. Expression of this cluster is rapidly downregulated upon differentiation, coincident with an elongation of the cell cycle. The miR-290 cluster maintains a very short cell cycle in ES by suppressing the G1/S restriction. A similar suppressed G1/S restriction is observed in cancer cells [54], and it is interesting to note that miR-106b promotes cell cycle progression in a breast cancer cell line [55], by mechanisms similar to the miR-290 cluster in ES, indicating similarities in the molecular control of the cell cycle of embryonic and cancer cells [56]. In our analysis, the predicted target genes for mmu-miR-294 were enriched in pathways related to MAPK-signaling, apoptosis, and cancer. A recent report found that the miRNAs-290 cluster was more abundantly expressed in the inner cell mass than in the trophectoderm of the blastocyst, and the authors suggest that this asymmetric pattern of stemness-controlling miRNA expression contributes to cell specialization [57].

Another gene found to be downregulated in developing embryos following paternal B[a]P exposure was mmu-miR-210. Controlling of the mitochondrial metabolism during hypoxia has been shown to involve miR-210, which is readily and dynamically affected by hypoxia inducible factor 1 (HIF-1*α*), the master regulator of the hypoxic response [58]. The cell cycle regulator E2F3 [59], the receptor tyrosine kinase ligand ephrin A3 [60], and the DNA repair protein RAD52 [61] have all been studied as targets for repression by miR-210. The iron-sulfur cluster assembly proteins ISCU1 and ISCU2 have been identified as direct targets for miR-210 in human lung endothelial cells [62]. Besides mitochondrial metabolism, the miR-210 may also be involved in other hypoxia-dependent processes such as iron metabolism, which is controlled by ISCU1/2 activity [63]. Furthermore, hypoxic repression of mitochondrial function by miR-210 and ISCU1/2 could theoretically trigger mitophagy—a form of autophagy in wihich defective mitochondria are selectively delivered to lysosomes for degradation. Mitophagy has been characterized as a-HIF-dependent mechanisms [64], but it remains to see if miR-210 itself induces mitophagy.

Among the genes that were downregulated, the most dramatic response was seen for mmu-miR-483. Aberrant expression of miR-483 has been reported in mice fed high-fat diets [65] indicating a role of this miRNA in energy metabolism. Consistent with the identification of miR-483-5p as an intronic miRNA encoded within the second intron of the Igf2 gene [66], a recent report confirms coexpression of miR-483-5p and insulin-like growth-factor 2 (Igf2) [67]. Igf2 has been primarily implicated in the regulation of prenatal growth [68], but mice carrying the Igf2 transgene have also been shown to have reduced bodyfat [69], and reduced Igf2 expression in adults have been shown to be accompanied by increased fat deposition [70]. In their report, Ma and coworkers found that overexpression of miR-483-5p lead to a decrease rather than an increase in Igf2 mRNA and the authors suggest that miR-483-5p regulates Igf2 expression both by suppressing Socs3 and via an additional undiscovered mechanism [67].

Another miRNA that was found to be downregulated in embryos of B[a]P exposed fathers relative to embryos of control fathers was miR-346. MiR-346 has been shown to be involved in regulation of carcinogenesis, inflammatory response, and differentiation [71–73]. Interestingly miR-346 has also been shown to target receptor-interacting protein 140 (RIP140) mRNA, and thereby upregulate its protein expression without altering mRNA levels [74]. RIP140 is a transcription coregulator that modulates the activities of many nuclear receptors and transcription factors [75]. The physiological function of RIP140 has been demonstrated in a gene knockout animal model and cell-line based systems; these include roles in gluoconeogenesis, glycolysis, fatty acid oxidation, and mitochondria biogenesis as well as modulation of hormone target genes [76–79]. The reduced expression of miR-346 observed in embryos of exposed fathers in the present study could indicate reduced RIP140 transrepression of gene regulation.

A conservative evaluation of up- and downregulated miRNAs in developing embryos is advisable due to the inherent complexity of these mechanisms. Still, the study of gene and miRNA expression profiles in developing embryos descending from exposed fathers, as exemplified in this report, could open up new avenues in our understanding of sublethal transgenerational effects of environmental exposure. Previous experiments looking at miRNA expression in developing embryos have pooled hundreds of mouse embryos [57]; here we demonstrate that miRNA expression profiles can be obtained from very few embryos. This illustrates a potential of similar experimental approaches in relation to the REACH EU Directive (Registration, Evaluation, Authorization and Restriction of Chemicals), implying that chemicals may be tested for effects on fertility and development using very few animals.

Our study has some limitations with respect to biological and technical replicates. Furthermore, mRNA expression profiling of the target genes from *in-vitro* fertilized embryos of B[a]P exposed fathers would have strengthened the findings. There may also be some incidences of false predictions, among the identified target genes. To reduce the likelihood of such errors, we narrowed and refined the list of target genes by comparing them with publically available gene expression data.

In conclusion, preconceptional paternal exposure to B[a]P may affect miRNA expression in the developing mouse embryo. More research is needed to fully appreciate the implications of early dysregulated miRNA expression, but given the wide array of cellular processes targeted by miRNAs, undesired consequences are expected. Gene and miRNAs expression in early embryos may provide valuable knowledge about potential transgenerational effect of sublethal exposure to exogenous compounds. 

## Supplementary Material

Supplementary Table 1: Complete list of the enriched KEGG pathway categories for target genes for the selected six up-regulated and the six down-regulated miRNA target genes. The table provides the number of reference genes in the category (C), number of genes in the gene set and also in the category (O), expected number in the category (E), Ratio of enrichment (R), p-value from hypergeometric test, and p-value adjusted by multiple test adjustment by the Benjamini & Hochberg method.
Supplementary Table 2: Complete list of the relative expression of 101 microRNAs data expressed in the Figure 2 heatmap, after filtering and normalization. The twelve most dysregulated genes are highlighted in bold italics.
Supplementary Figure 1: The twelve most dysregulated miRNAs in the present experiment and their typical expression in various mouse tissues, obtained from the miRz database (http://www.mirz.unibas.ch/). Yellow samples represent up-regulated miRNAs whereas blue samples represent down-regulated miRNAs. Black means not detected.

## Figures and Tables

**Table 1:** Expression levels of the six up- and down-regulated miRNAs in developing embryos derived from B[a]P exposed fathers, relative to unexposed controls.

miRNA	Direction	2-cell	8-cell	Blastocyst	# target genes^(a) ^
mmu-miR-574-5p	Up	2.73	2.00	1.91	531
mmu-miR-467f	Up	5.83	1.23	2.23	972
mmu-miR-294	Up	3.22	1.74	1.19	646
mmu-miR-210	Up	2.27	1.26	2.48	127
mmu-miR-197	Up	1.91	2.41	1.80	379
mmu-miR-139-3p	Up	4.33	2.56	1.93	333

mmu-miR-99a	Down	−1.29	−2.56	−0.56	64
mmu-miR-483	Down	−0.39	−1.23	−13.45	107
mmu-miR-466f-5p	Down	−4.40	−2.86	−2.08	318
mmu-miR-346	Down	−4.16	−2.04	−0.32	453
mmu-miR-1906	Down	−4.63	−1.38	−1.03	615
mmu-miR-1896	Down	−6.18	−4.94	−3.06	638

Note: Log2-transformed ratio of B[a]P and control; ^(a) ^predicted targets.

**Table:** (a) Six upregulated miRNAs

Enriched KEGG pathways	Selected dysregulated miRNAs					
	mmu-miR-139-3p	mmu-miR-197	mmu-miR-210	mmu-miR-294	mmu-miR-467f	mmu-miR-574-5p
Metabolic pathways	37	27	8	32	47	28
Cytokine-cytokine receptor interaction		7		11	23	
Insulin signaling pathway				9	14	11
Pathways in cancer				16	22	15
Regulation of actin cytoskeleton		6		16		13
Apoptosis		4		8		
MAPK signaling pathway				16		13
Pancreatic cancer			2			8

**Table:** (b) Six downregulated miRNAs

Enriched KEGG pathways	Selected dysregulated miRNAs					
	mmu-miR-1896	mmu-miR-1906	mmu-miR-346	mmu-miR-466f-5p	mmu-miR-483	mmu-miR-99a
Metabolic pathways	54	40	23	7	7	7
Pathways in cancer		14	10	4		2
Cell cycle				3	2	2
Neuroactive ligand-receptor interaction		13	9	5		
TGF-beta signaling pathway			6	3	2	
Calcium signaling pathway	12	11				
Chemokine signaling pathway	11				2	
Glycerophospholipid metabolism		7				3
Leukocyte transendothelial migration	8	9				
Melanogenesis				3	2	
Progesterone-mediated oocyte maturation			5		2	

Note: number of target genes for particular miRNA that are enriched in particular KEGG pathway.
