# Supplementary material for: Paternal Benzo[a]pyrene Exposure Modulates MicroRNA Expression Patterns in the Developing Mouse Embryo
Source: Int J Cell Biol. 2012 Apr 4;2012:407431. doi: 10.1155/2012/407431 (PMC3324892; doi:10.1155/2012/407431)
Supplement: Supplementary file 1 — Supplementary Table 1: Complete list of the enriched KEGG pathway categories for target genes for the selected six up-regulated and the six down-regulated miRNA target genes. The table provides the number of reference genes in the category (C), number of genes in the gene set and also in the category (O), expected number in the category (E), Ratio of enrichment (R), p-value from hypergeometric test, and p-value adjusted by multiple test adjustment by the Benjamini & Hochberg method. Supplementary Table 2: Complete list of the relative expression of 101 microRNAs data expressed in the Figure 2 heatmap, after filtering and normalization. The twelve most dysregulated genes are highlighted in bold italics. Supplementary Figure 1: The twelve most dysregulated miRNAs in the present experiment and their typical expression in various mouse tissues, obtained from the miRz database (http://www.mirz.unibas.ch/). Yellow samples represent up-regulated miRNAs whereas blue samples represent down-regulated miRNAs. Black means not detected. [file 407431.f1.doc]

Supplementary table 1.
